# Medical Institute of Louisville

**Published:** 1843-11

**Authors:** 


### MEDICAL INSTITUTE OF LOUISVILLE.

Students are coming in well to this institution, and the class promises to be one of the largest that has yet attended it.

